# Indirect Role of Sprint Performance in the Relationship Between Explosive Power and Change-of-Direction Ability in Adolescent Athletes: A Structural Equation Modeling Study

**DOI:** 10.3390/sports14060217

**Published:** 2026-05-26

**Authors:** Ying-Fang Liu, Huan-Chieh Chen, Tso-Yen Mao

**Affiliations:** 1 Department of Health and Leisure Management, Hsin Sheng Junior College of Medical Care and Management, Taoyuan 32544, Taiwan; kghs@hsc.edu.tw; 2 Center for General Education, National United University, Miaoli 360302, Taiwan; 898300199@nuu.edu.tw; 3 Department of Leisure Services Management, Chaoyang University of Technology, Taichung 41349, Taiwan

**Keywords:** explosive power, sprint performance, mediation, neuromuscular capacity, structural equation modeling

## Abstract

Background: This study examines the hierarchical determinants of change-of-direction (COD) ability in adolescent athletes to assess whether sprint performance functions as a potential intermediate variable of the relationship between explosive power and COD performance. Methods: The study recruited 86 high school athlete participants. Explosive power was assessed using the countermovement jump (CMJ) and squat jump (SJ). Sprint performance was evaluated using 10 m and 20 m sprint tests. COD performance was assessed using the 505 pre-planned COD test. Body composition was also recorded. Results: Correlation analyses indicates that 20 m sprint time was positively associated with COD performance (r = 0.67–0.82), whereas CMJ performance was significantly and negatively associated with COD performance (r = −0.65 to −0.68). Multiple regression analysis explained 63.6% of the variance in COD performance; sprint performance emerged as the strongest predictor. Indirect effect analysis showed that the effect of explosive power on COD performance is consistent with an indirect pathway by sprint performance. Structural equation modeling supported a hierarchical model: Explosive power was associated with sprint performance, which in turn was associated with COD ability. Conclusions: The findings suggest that improvements in COD performance among adolescent athletes may depend on enhancing explosive power while optimizing acceleration and speed-transfer capacity. These findings provide practical implications for athlete selection and training program design in youth sports.

## 1. Introduction

COD ability is a key determinant of athletic performance in many field and court sports. In association football, COD performance is closely linked to short-distance sprint capacity and explosive braking capability, which underpin match-relevant actions such as rapid directional changes, accelerations, and decelerations [1], comprising the fundamental basis of dribbling. In basketball, athletes frequently change direction during offensive and defensive maneuvers, making COD an essential component of on-court athleticism [2]. In racket sports such as badminton, COD ability is closely associated with explosive, multidirectional movement demands that characterize high-level play [3]. In collision sports such as rugby and American football, agility—defined as the ability to respond rapidly to unforeseen events—is a critical performance component that integrates motor and cognitive elements [4]. Notably, directional quickness and COD performance are not solely reducible to isolated physical qualities; emerging evidence shows that perception–action coupling and neuromuscular responsiveness also contribute to quickness and movement regulation in team sport athletes [5]. Given the ubiquity of directional changes across these sports, understanding the underlying determinants of COD performance is key for athlete development and training optimization [6].

Explosive power plays a well-established role in COD performance, often assessed using lower-limb metrics such as CMJ and SJ. Evidence consistently indicates moderate-to-strong relationships between vertical jump performance and COD ability. For instance, Papla et al. found significant negative correlations between CMJ height and modified T-agility times (a pre-planned COD measure; r = −0.69) among male basketball players [7]. França et al. (2024) found a moderate correlation between SJ and T-test COD performance (r = −0.47) among youth soccer players [8]. Yamashita et al. (2024) showed that CMJ height was the sole predictor of 10 m pro-agility performance (adjusted R^2^ = 0.234) among female athletes [9]. Burke et al. (2023) found that vertical jump mediated the effect of maximum strength on COD performance in collegiate American football players (β = −0.376, *p* < 0.001) [10]. However, the relationship between explosive power and COD performance seems inconsistent across populations and testing protocols. For instance, Stanković et al. (2022) reported weak, non-significant correlations between jump measures and several COD tests in elite female soccer players [11]. Comparatively, Kadlubowski et al. (2021) found that SJ performance had a limited and task-dependent influence on COD ability among elite youth soccer players (r ranging from −0.02 to −0.29) [12]. Inconsistent findings imply that the mechanisms connecting explosive power to COD performance may be more complex than previously assumed. The findings highlight the need for further inquiry into potential indirect and moderating factors that govern this relationship.

Although the link between explosive power and COD is well established, current research indicates that sprint performance may serve as an intermediate pathway. Specifically, linear sprint performance, especially over short distances (10–20 m), demonstrates strong predictive value for COD ability. For instance, Zhang et al. (2022) found that 10 m sprint time was strongly associated with 505 COD performance (effect size = 0.64–0.71), and emerged as a primary determinant in multiple regression models in addition to maximal deceleration power [13]. Baena-Raya et al. (2021) found very strong correlations between sprint mechanical outputs, specifically maximal velocity (V_0_) and maximal horizontal power (Pmax), and COD performance (r = −0.767 and −0.821, respectively, *p* < 0.01) [14]. These findings suggest that sprint force–velocity characteristics are more strongly related to COD than traditional jump-based power measures. Furthermore, Kadlubowski et al. (2021) demonstrated that linear sprint performance explained the greatest variance in COD ability across multiple tests (r^2^ = 0.18–0.39), surpassing the explanatory power of SJ and maximal strength measures [12]. The above findings indicate a hierarchical model in which explosive power is associated with sprint performance, subsequently determining COD ability (power → sprint → COD). However, formal mediation analyses examining sprint performance as a mediating variable between explosive power and COD remain limited, particularly regarding adolescent populations. Based on the above, the present study tested whether a speed-mediated association exists and whether the observed relationships are consistent with a hierarchical framework that may inform more targeted training interventions for enhancing COD performance in youth athletes.

Despite growing interest in the determinants of COD performance, a critical methodological limitation persists in the current research: the predominance of correlational, cross-sectional study designs that cannot establish causal or mechanistic relationships. Notably, although structural equation modeling (SEM) and mediation analysis can be used to test theoretically plausible pathways, when applied to cross-sectional data, they do not establish causal relationships. In a recent systematic review, Singh et al. (2024) noted that most studies examining biomechanical determinants of COD have relied on correlational analyses: “the impact of moderating factors on COD performance was minimally examined” [15]. Beyond physical qualities such as strength and explosiveness [16,17], the technical execution of COD movements represents a critical determinant, as biomechanical inefficiencies and technical errors during directional changes can directly compromise COD performance outcomes [18,19]. Thus, greater emphasis on the biomechanical and technical dimensions of COD is warranted. Moreover, Yamashita et al. (2024) caution that “correlation does not imply causation, and potential confounders may affect the correlation” when interpreting relationships between jump performance and COD ability [9]. Reliance on bivariate associations limits understanding of the mechanisms by which physical qualities affect sport-specific performance. Moreover, Novak et al. (2021) indicate that performance research has “frequently involved multiple univariate analyses.” They demonstrated how path analysis and SEM can “enhance the mechanistic interpretation of key performance indicators” by providing a multivariate, mechanistic perspective [20].

**Figure 1:**
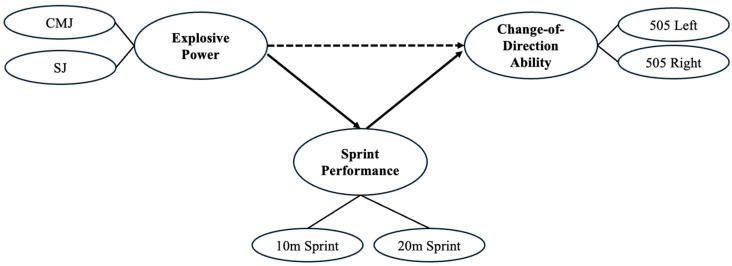
Hypothesized structural model of the relationships among explosive power, sprint performance, and COD ability in adolescent athletes. Explosive power is modeled as a latent variable defined by CMJ and SJ, while sprint performance is captured by 10 m and 20 m sprint times. COD ability is measured using the 505 change-of-direction test in both turning directions. The model specifies a hierarchical structure in which explosive power is hypothesized to be associated with sprint performance, which in turn is associated with COD ability. A dashed path indicates a potential direct effect of explosive power on COD ability, suggesting partial mediation.

Based on the above, applications of SEM in sports science may help elucidate hierarchical relationships among physical qualities. Specifically, Burke et al. (2023) employed multigroup path analysis to demonstrate that vertical jump fully mediated the relationship between maximal strength and COD performance in collegiate American football players [10]. Domaradzki et al. (2021) applied mediation analysis to illustrate that COD speed mediated the relationship between sport type and reactive agility in elite female athletes (noting that reactive agility, unlike pre-planned COD ability assessed in the present study, incorporates perceptual and decision-making components) [21]. The findings highlight the potential value of advanced statistical modeling in extending simple associations to test mechanistic pathways. Yet, despite these methodological advances, formal mediation or SEM analyses examining the hierarchical power → sprint → COD pathway in adolescent populations remain limited. As such, the present study employed SEM and mediation analysis to test whether sprint performance mediates the relationship between explosive power and COD ability among adolescent athletes (Figure 1).

## 2. Materials and Methods

### 2.1. Participants

The study recruited high school student athletes from public and private senior high schools in Taoyuan City, Taiwan. A total of 86 adolescent athletes (49 males, 37 females) voluntarily participated. Coaches of school representative teams assist with recruitment by informing eligible athletes about the study. All participants are active team members with regular training and competitive experience.

The study sample encompassed participants from multiple sports disciplines, including badminton, taekwondo, handball, basketball, and kabaddi. Participants were engaged in regular training, with approximately 4–6 sessions per week. The average training experience was 4.2 ± 1.7 years, indicating that most participants had engaged in structured training since early adolescence. The mean age of the participants was 16.2 ± 0.9 years.

Physical performance testing was conducted at public sports centers and specialized athletic facilities within participating schools. To ensure measurement reliability and consistency, trained research personnel administer all assessments in accordance with standardized testing protocols.

Ethical clearance for this study was granted by the Research Ethics Committee of National Chengchi University (Approval No. NCCU-REC-202205-E060). The study adhered to established ethical principles for research involving human participants. Before participation, written consent was secured from both the participants and their guardians. The study was conducted in accordance with the Declaration of Helsinki. Detailed participant characteristics are presented in Table 1.

### 2.2. Study Design

A cross-sectional design was adopted in this study to examine the relationships among lower-limb explosive power, linear sprint performance, and COD ability in adolescent athletes [22,23]. This design was intended to examine associations among variables within a theoretically grounded framework, rather than to establish causal relationships. The testing battery comprised assessments of lower-limb explosive power (countermovement jump [CMJ] and squat jump [SJ]), linear sprint performance (10 m and 20 m), and COD ability was assessed using the 505 pre-planned change-of-direction test, with both left- and right-turning directions recorded. To maintain consistency in the testing environment and measurement protocols, standardized testing procedures were followed; all assessments were conducted by trained research personnel.

To reduce injury risk and ensure stable performance, participants completed a standardized warm-up that included light jogging and dynamic stretching before testing. To limit fatigue effects on subsequent measurements, testing sessions were arranged in a predetermined sequence consistent with established testing principles [24,25,26]. Participants completed CMJ testing to assess lower-limb explosive power, followed by SJ testing to evaluate concentric power, given that vertical jump measures are highly sensitive to neuromuscular fatigue and are therefore conducted before more metabolically demanding tasks [27]. To assess short-distance acceleration and speed capacity, linear sprint performance was evaluated using 10 m and 20 m sprint tests. A rest interval of approximately 3–5 min was provided between tests to minimize fatigue accumulation and maintain performance reliability [27,28].

### 2.3. Physical Performance Tests

Testing was carried out at public sports centers and designated indoor school facilities. All assessments were administered by trained personnel using standardized procedures. Participants completed a structured warm-up of light jogging and dynamic stretching before testing to reduce injury risk and ensure consistent performance. Performance data were captured using electronic timing gates and validated jump measurement devices to ensure accuracy and reliability. Rest intervals of approximately 3–5 min were scheduled between tests to limit fatigue effects. Each test was repeated three times, and the best result was selected for analysis.

#### 2.3.1. Explosive Power: CMJ and SJ

Lower-limb explosive power was evaluated using CMJ and SJ tests. Jump height was measured with a GymAware RS (Kinetic Performance Technology, Canberra, Australia; linear position transducer, LPT) [29,30]. The device was operated using the GymAware V2 mobile application (iPad-based interface) for real-time monitoring and data acquisition. The GymAware RS has demonstrates moderate-to-high test–retest reliability for both CMJ (ICC = 0.95, CV = 0.74%) and SJ (ICC = 0.84, CV = 3.25%), supporting its use as a reliable instrument for measuring jump height in athletic populations [31,32]. Participants stood with their feet positioned shoulder-width apart and kept their hands on their hips throughout testing to reduce the influence of arm swing. During the CMJ, participants executed a rapid downward movement immediately followed by a maximal vertical jump. For the SJ, participants adopted a static squat position with approximately 90° knee flexion for 1–2 s before jumping vertically, minimizing the contribution of the stretch–shortening cycle. Each participant completed three trials for CMJ and SJ, with the highest jump height selected for analysis. Before testing, the GymAware RS device was placed directly on the ground beneath the participant; the tether was secured to a belt at the participant’s waist. Tether alignment was carefully checked to ensure vertical positioning, thereby reducing measurement errors caused by horizontal displacement. Before each trial, a zero-height calibration procedure (“zeroing”) was performed with the participant standing upright to define the reference position for displacement measurement. The application was set to automatic jump detection mode. Since the system is factory-calibrated and periodically maintained according to manufacturer guidelines, no additional device calibration was required during testing.

#### 2.3.2. Sprint Performance: 10 m and 20 m Sprint

Linear sprint performance was evaluated using 10 m and 20 m sprint tests. Sprint times were measured with infrared electronic timing gates (ATIS E01-631B-Y02, ATIS Technology Co., Ltd., Taipei, Taiwan). Participants began from a stationary standing position behind the starting line and initiate the sprint in a self-initiated manner, with timing triggered when the participant’s torso passes through the initial timing gate.

Timing gates were placed at the starting line, 10 m, and 20 m to measure short-distance acceleration and sprint performance [33,34]. The timing gate sensors were standardized at approximately 100 cm in height, corresponding to the participant’s trunk level, to minimize potential interference from limb movement and ensure measurement consistency. This approach ensured that timing was triggered by torso passage rather than limb movement, decreasing the likelihood of premature or repeated signal activation.

Participants completed a 10 min dynamic warm-up, including 2–3 progressive sprint trials at approximately 50–80% of maximal effort before testing. During testing, participants were instructed to sprint maximally through the finish line and to avoid decelerating before crossing the final timing gate.

Each participant completed three trials, with 5 min rest intervals between trials to allow for adequate recovery of the ATP–PCr energy system. The fastest time was recorded for subsequent analysis.

#### 2.3.3. COD Ability: 505 Test

COD ability was evaluated using a 505 Test. Total completion time was recorded using infrared electronic timing gates (ATIS E01-631B-Y02, ATIS Technology Co., Ltd., Taipei, Taiwan). Compared to the traditional 505 protocol, the modified version was conducted without a flying start, with timing initiated directly from the starting line, which better isolates braking and re-acceleration performance during the COD task.

The test was conducted over a distance of 5 m. The starting/finishing line (0 m) was equipped with timing gates; a turning line was marked at 5 m. A cone was placed beyond the turning line as a visual guide without obstructing foot placement. Participants adopted a stationary standing start, positioning the lead foot just behind the starting line. Timing begins as the torso crosses the first timing gate, and participants accelerate maximally toward the turning line.

At the 5 m mark, participants plant the outside foot on or beyond the line, perform a rapid 180° turn, and sprint back to the start. Timing ends when the torso crosses the start/finish gate on return. Sensors are placed at trunk height (~100 cm) to standardize measurement, limit limb interference, and prevent premature triggering.

COD performance is evaluated in both left- and right-turning directions, with three trials conducted per condition. Participants rest for 3–5 min between trials to reduce fatigue effects. The fastest performance from each direction is retained for analysis.

The 505 Test is frequently used in sports performance research to evaluate an athlete’s pre-planned COD ability, specifically the capacity to decelerate, change direction, and re-accelerate during high-speed movements. As a pre-planned protocol, it does not capture the perceptual or reactive components associated with reactive agility.

### 2.4. Anthropometric Measures

Body composition was assessed prior to physical performance tests using a bioelectrical impedance analyzer (Dial H30/H30NWi, InBody Co., Ltd., Seoul, Republic of Korea) [35]. The primary variables included body fat percentage and skeletal muscle mass. Participants were tested wearing light sports clothing and standing barefoot on the analyzer platform, following manufacturer guidelines. To control for hydration and metabolic variability, participants were instructed to abstain from vigorous exercise, large meals, and caffeine for at least 12 h before testing. Bioelectrical impedance analysis (BIA) estimates body composition by applying a low-level electrical current through body tissues to calculate indices such as body fat percentage and muscle mass. This method is non-invasive, quick to administer, and widely used in sports science and physical fitness research. Measurements were performed by trained research personnel following standardized procedures to ensure accuracy and consistency.

### 2.5. Statistical Analysis

All raw data were organized and screened using Microsoft Excel (Version 16.104, Microsoft Corp., Redmond, WA, USA), including data cleaning, verification of missing values, and standardization of variable formats. After preprocessing, statistical analyses were conducted in the Python programming environment using Jupyter Notebook (Version 7.3.2) within the Anaconda distribution (Anaconda Navigator Version 2.7.0). Descriptive statistics were computed for all variables and presented as mean ± standard deviation (SD). Variables included anthropometric measures and physical performance indicators, such as height, body mass, body fat percentage, muscle mass, CMJ height, SJ height, 10 m sprint time, 20 m sprint time, and 505 COD test performance. Before inferential analyses, the normality of all continuous variables was examined using the Shapiro–Wilk test. Maximum Likelihood Estimation (MLE) was adopted as the SEM estimator because it is the standard approach for continuous, normally distributed indicators [36,37]. SEM analyses were conducted using a latent variable framework, in which mediation effects were estimated at the latent construct level. Pearson’s correlation analysis was conducted to assess the associations among the physical performance variables. Multiple regression analysis was conducted to examine the associations between explosive power and sprint performance on COD, and to examine the relationships among lower-limb explosive power, sprint performance, and change-of-direction ability. Variance inflation factors (VIF) were calculated to assess potential multicollinearity among the independent variables.

Moreover, SEM was used to examine the hierarchical relationships among explosive power, sprint performance, and COD performance. The proposed model comprised three latent constructs: power, sprint, and COD. CMJ and SJ performance represented the power construct, the sprint construct was represented by 10 m and 20 m sprint times, and the COD construct was represented by left and right 505 test performances. The adequacy of the sample size for SEM was assessed before analysis. While Monte Carlo simulation guidelines generally recommend a minimum of N ≥ 200 for stable SEM estimation [38], the present sample (N = 86) is comparable to those reported in similar field-based studies conducted in adolescent sport populations. Additionally, the relatively simple model structure (three latent constructs, six observed indicators) decreases the parameter-to-sample-size ratio, which partially mitigates concerns regarding sample size adequacy [37]. No post hoc model modifications (e.g., based on modification indices) were conducted, and the hypothesized model was retained to maintain theoretical interpretability. Bootstrapping is also used to improve the robustness of indirect effect estimates under the given sample size conditions [39]. These were specifically applied to the SEM-based mediation model to estimate indirect effects at the latent construct level. Model fit was evaluated using multiple indices, including the comparative fit index (CFI), Tucker–Lewis Index (TLI), Goodness-of-Fit Index (GFI), Root Mean Squared Error of Approximation (RMSEA), and Standardized Root Mean Square Residual (SRMR). Acceptable model fit was defined based on the widely adopted criteria proposed by Hu and Bentler (1999) [40]: CFI ≥ 0.95 and TLI ≥ 0.95 indicate a good fit, with values ≥ 0.90 considered acceptable by conventional standards [41]; RMSEA ≤ 0.06 indicates close fit; and SRMR ≤ 0.08 is considered acceptable [40]. GFI values ≥ 0.90 were additionally used as a supplementary indicator of acceptable fit.

Bootstrapping is used to examine indirect effects and determine whether sprint performance mediates the relationship between explosive power and COD performance. A resampling procedure with 5,000 iterations generates bias-corrected 95% confidence intervals, since it does not require normality assumptions and yields more robust mediation estimates than traditional normal-theory approaches [39,42]. Indirect effects were estimated using repeated resampling procedures to generate confidence intervals for the mediation effect. Indirect effects were considered statistically significant when the 95% confidence interval did not include zero. Statistical significance for all analyses was set at *p* < 0.05.

## 3. Results

### 3.1. Descriptive Statistics

Table 1 presents the descriptive statistics for anthropometric and physical performance variables. A total of 86 adolescent athletes were included in the analysis. The observed values generally align with previously reported ranges for adolescent athletes. Notably, variability was observed across key performance indicators, particularly in sprint times and COD performance, suggesting heterogeneity in acceleration and directional change capacities within the sample.

### 3.2. Correlation Analysis

Table 2 presents the Pearson’s correlation coefficients among lower-limb explosive power, sprint performance, and COD ability. Significant correlations were observed among all performance variables (*p* < 0.01). Explosive power (CMJ and SJ) was negatively correlated with sprint times and COD times (r = −0.63 to −0.77, *p* < 0.01). This finding indicates that greater jump performance was associated with faster sprinting and COD performance. Sprint times were positively correlated with COD times (r = 0.63 to 0.82, *p* < 0.01), indicating consistent associations across tasks.

A very strong correlation was observed between short-distance sprint measures (10 m and 20 m; r = 0.97, *p* < 0.01). This finding suggests high consistency across sprint performance metrics. Given this high correlation, the variables may reflect substantial shared variance. Nevertheless, both measures were retained to represent sprint performance across slightly different acceleration phases, thereby supporting the construct validity of the latent sprint factor rather than indicating problematic redundancy.

### 3.3. Regression Analysis

Multiple regression analysis was conducted to examine the predictors of COD performance (Table 3). The regression model explained a substantial proportion of the variance in COD performance (R^2^ = 0.636, adjusted R^2^ = 0.626). Sprint performance (20 m sprint time) emerged as the strongest predictor of COD performance, with longer sprint times associated with poorer COD performance.

While both CMJ and SJ were initially considered as indicators of lower-body explosive power, only CMJ was retained in the final regression model due to the high correlation between them (r = 0.80); including both simultaneously could introduce multicollinearity. Moreover, CMJ had a stronger and more consistent association with COD performance, supporting its use as a representative indicator of explosive power. CMJ was negatively associated with COD performance, indicating that greater CMJ power was associated with better COD outcomes.

Indirect effect analysis suggested that the association between explosive power and COD performance may be partially explained by sprint performance. Variance inflation factors (VIFs) were 2.44 for both 20 m sprint time and CMJ, remaining well below the commonly accepted threshold (VIF < 5), indicating no evidence of multicollinearity.

### 3.4. SEM

**Figure 2:**
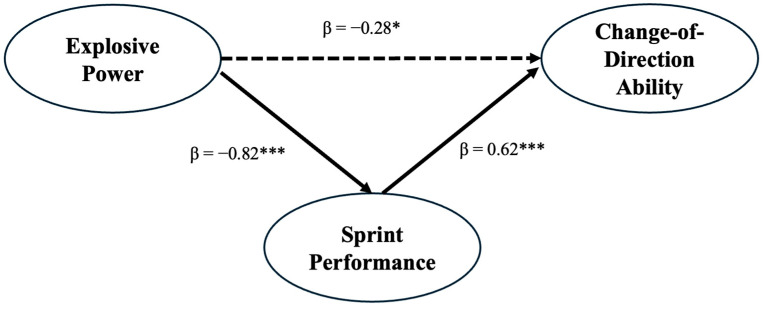
Structural equation model depicting the hierarchical relationships among explosive power, sprint performance, and COD ability. Standardized path coefficients (β) are shown along the arrows. Solid lines represent statistically significant paths, whereas the dashed line represents the direct path from explosive power to COD ability. * *p* < 0.05, *** *p* < 0.001.

The SEM results are consistent with a hierarchical association among explosive power, sprint performance, and COD ability (Table 4; Figure 2). Explosive power was significantly associated with sprint performance (β = −0.82, *p* < 0.001), which in turn was significantly associated with COD ability (β = 0.62, *p* < 0.001). The direct path from explosive power to COD ability remained significant (β = −0.28, *p* = 0.043). This finding indicates that both direct and indirect associations may be present, aligning with a partial mediation pattern.

The measurement model showed adequate factor loadings for the observed variables representing each latent construct. Overall model fit indices indicate acceptable-to-good fit (CFI = 0.967, TLI = 0.917, SRMR = 0.060), even though the RMSEA was relatively high (0.196).

## 4. Discussion

The present study examined the hierarchical determinants of COD ability in adolescent athletes. It assessed whether sprint performance may act as an intermediate factor linking explosive power and COD performance. Sprint performance was identified as the strongest correlate of COD performance, with 20 m sprint time exhibiting a very large association with 505 COD performance. This finding is consistent with accumulating evidence across multiple sport contexts. Similarly, Gisladottir et al. (2024) reported an extremely large correlation (r = 0.90) between the 20 m sprint and the modified agility T-test (a pre-planned COD measure) in senior professional team sport players [43]. Furthermore, Baena-Raya et al. (2021) found very strong correlations between sprint mechanical outputs (maximal velocity V_0_ and maximal horizontal power Pmax) and 505 COD times (r = −0.767 and −0.821, respectively) among female futsal players [14]. In adolescent populations, Loturco et al. (2022) also demonstrated large to very large correlations (r = 0.62–0.84) between linear sprint velocity and COD ability in young badminton players [44]. Furthermore, Zhang et al. (2022) reported that 10 m sprint time was a primary variable strongly associated with 505 COD performance (effect size = 0.64–0.71) in soccer players [13]. These findings collectively suggest that sprint performance is closely associated with COD ability, particularly in pre-planned COD tasks.

However, COD performance is a multifactorial construct that goes beyond sprint and propulsive capabilities. Alongside sprint ability, braking and deceleration capacities play a central role in COD performance. Athletes with higher COD proficiency tend to show greater peak horizontal braking forces and more efficient velocity reduction at the penultimate and final foot contacts during 180° turning tasks [18]. Movement techniques and re-acceleration strategies are also associated with COD outcomes; evidence demonstrates that foot placement, whole-body and pelvic rotation toward the exit direction, and center of mass (COM) lowering at the penultimate contact are associated with faster completion times and improved exit velocity [18,45]. Inter-limb asymmetry has also been suggested as an additional factor, as performance and kinematic differences between limbs may influence COD execution and should be considered when interpreting limb-specific test scores [46]. Moreover, directional quickness and sport performance may not be fully captured by isolated force and sprint metrics alone; it may also be shaped by task-specific and perception–action demands. Recent intervention-based evidence shows that training approaches targeting perception–action coupling may improve quickness and neuromuscular responsiveness in team sport athletes, supporting the notion that COD-related performance encompasses both physical and perceptual–cognitive dimensions [5]. These findings suggest that while the hierarchical power → sprint → COD pathway identified in the present study provides a useful conceptual framework, a more comprehensive understanding of COD performance likely requires integrating biomechanical, technical, asymmetry-related, and perception–action factors alongside force production metrics.

The indirect effect analysis in the present study suggests that sprint performance may function as an intermediate variable linking explosive power (CMJ) and COD ability, as evidenced by a 95% confidence interval for the indirect effect that excluded zero. However, given the study’s cross-sectional design, this finding should be interpreted as a statistical association consistent with an indirect effect pattern rather than definitive evidence of a causal mechanism. This finding aligns with past research indicating a potential speed-related pathway linking explosive power to COD performance in adolescent athletes. The SEM results are consistent with a hierarchical association pattern aligned with the proposed theoretical framework, suggesting that explosive power is statistically associated with sprint performance, which in turn is statistically associated with COD ability. These associations do not, however, imply a direct causal mechanism, as the cross-sectional design precludes causal inference. This hierarchical relationship aligns with the theoretical frameworks proposed in recent studies [12,47]. The findings indicate that sprint performance explains greater variance in COD ability than isolated jump measures. Specifically, Kadlubowski et al. (2021) reported that linear sprint explained 18–39% of COD variance among elite youth soccer players [12], while SJ exhibited lower and sometimes negative explained variance. This finding indicates that sprint capacity may represent a more proximal factor associated with COD performance than explosive power alone. The above findings suggest a potential intermediary role of sprint performance in linking neuromuscular power and sport-specific directional change capacity in youth athletes.

The hierarchical relationship observed in this study, in which explosive power is associated with sprint performance, which in turn relates to COD Ability, can be partially explained by the biomechanical transfer of neuromuscular power to horizontal force production during acceleration. Neuromuscular power, assessed via vertical jump tests (countermovement jump [CMJ] and squat jump [SJ]), reflects the capacity of the lower-limb musculature to generate high rates of force development and concentric power output [48]. However, the transfer of this neuromuscular capacity to sprint performance may depend on the athlete’s ability to orient force horizontally during ground contact. Specifically, Junge et al. (2021) found that while vertical jump power is associated with horizontal sprint capacity, exercises and tests highlighting horizontal force vectors (e.g., hip thrusts, horizontal jumps) provide greater transfer to sprint force production (F_0_) and early acceleration than purely vertical assessments [49]. This force–orientation specificity is considered critical because sprint acceleration is primarily influenced by maximal horizontal power (Pmax) and the distribution of force along the force–velocity profile, with higher theoretical maximal horizontal force (F_0_) being particularly advantageous for short-distance acceleration [48].

The present findings align with this proposed framework: athletes with greater explosive power (as indicated by CMJ and SJ performance) may be better able to generate horizontal propulsive forces during the acceleration phase of sprinting, which, in turn, may be associated with superior COD performance. Furthermore, the biomechanical determinants of this transfer are likely to operate at both the joint and whole-body levels. For instance, Sado et al. (2023) demonstrated that hip extensor-driven front-thigh rotation accounts for the majority (~55%) of horizontal external power generation during the sprint start, linking lower-limb concentric power production to horizontal propulsion at ground contact [50]. Trunk rotator musculature has been suggested to play a key role at the whole-body level. These muscles are not only essential for generating rotational torque to drive thorax–pelvis rotation during directional changes [51], but also contribute to the dynamic stabilization of the spine against sudden rotational perturbations during high-velocity maneuvers [52]. Moreover, effective trunk rotation has been shown to facilitate the reorientation of the COM toward the intended direction, with faster performers demonstrating greater internal pelvic and trunk rotation aligned with the exit direction [18,19]. In contrast, insufficient trunk strength may lead to inefficiencies in force transmission within the kinetic chain, potentially lowering COD speed and overall biomechanical efficiency [53].

During the acceleration phase, athletes with greater neuromuscular power are likely to generate larger net propulsive impulses, defined as the product of horizontal force and contact time; this may facilitate faster velocity gains and support both rapid linear acceleration and controlled deceleration required for effective directional changes [54]. Furthermore, Zhang et al. (2022) found that both 10 m sprint time (reflecting early acceleration capacity) and maximal deceleration power were primary determinants of 505 COD performance in soccer players, underscoring the dual role of horizontal force production in both propulsive and braking phases of COD tasks [13]. Thus, the hierarchical association pattern identified in the present SEM analysis is consistent with an underlying biomechanical cascade, though this interpretation remains mechanistic and inferential rather than directly evidenced by the present data. Neuromuscular power appears to represent a foundational capacity for force generation that may need to be directed horizontally to produce sprint acceleration. This acceleration ability, involving both propulsion and braking, is statistically associated with rapid COD performance in the present sample. These mechanisms have practical implications for training, indicating that enhancing horizontal force production and acceleration mechanics may yield greater improvements in COD performance than isolated vertical jump training in adolescent athletes.

Practically, the findings suggest that training programs should emphasize acceleration and sprint development to enhance COD performance among adolescent athletes. Since sprint performance appears to serve as a key mediator between explosive power and COD capacity, coaches and practitioners should prioritize training modalities that target horizontal force production and acceleration mechanics. In addition to sprint-focused training, practitioners should integrate COD technique modification, deceleration training, and asymmetry correction, as these factors may independently contribute to COD performance and may not be fully addressed through sprint and power training alone [45,46]. Acceleration drills, particularly when combined with plyometric exercises, have been shown to produce substantial improvements in sprint and COD performance in youth populations. For instance, Aloui et al. (2022) found that an eight-week combined plyometric and short-sprint training program (two sessions per week) in U-15 male soccer players yielded effect sizes of d ≥ 0.64 for 10 m and 30 m sprint times and d ≥ 0.66 for COD performance [55]. Furthermore, Niering et al. (2025) reported that a 20-week intervention combining plyometric drills immediately followed by maximal sprints generated improvements of 6.7% at 5 m, 4.8% at 10 m, and 3–4% in COD ability [56].

Several methodological considerations should be acknowledged. First, it is acknowledged that this level of inter-indicator correlation raises legitimate concerns regarding redundancy within the latent sprint construct. Nevertheless, both measures were retained for the following methodological reasons. The 10 m sprint primarily captures initial acceleration capacity (i.e., the ability to generate force rapidly from a stationary start), whereas the 20 m sprint reflects more sustained acceleration and the attainment of higher running velocities over a longer distance; these represent theoretically distinct, albeit closely related, phases of the sprint continuum [48,49]. Within a latent variable framework, high inter-indicator correlations are not inherently problematic, as the shared variance is explicitly modeled as the common factor, while residual variances remain separable [36]. Retaining both indicators strengthens the reliability and representational breadth of the latent sprint construct, consistent with the construct validity principle in SEM [36,37]. Nonetheless, it is recognized that this choice may limit the discriminant validity of the sprint factor, and future studies with larger samples may consider evaluating whether a single sprint indicator yields comparable model fit. Second, although the RMSEA was notably high (0.196), substantially exceeding the recommended threshold of ≤0.06 [40,41]. and this should be acknowledged as a meaningful limitation of the present model. It is well established that RMSEA is particularly sensitive to models with small degrees of freedom and limited sample sizes, which can produce inflated values even when the model structure is theoretically sound [40,41]. Accordingly, the overall model fit cannot be considered unequivocally acceptable, and the elevated RMSEA warrants caution when interpreting the robustness of the SEM results. Greater emphasis was therefore placed on CFI, TLI, and SRMR, which collectively suggest acceptable fit; however, the elevated RMSEA implies that the present findings should be regarded as preliminary and interpreted with appropriate caution, particularly given the modest sample size (N = 86).

The study limitations are as follows. First, the cross-sectional design limits the ability to draw causal inferences regarding relationships among explosive power, sprint performance, and COD performance. Even though the mediation analysis and SEM provide evidence supporting a hierarchical relationship, longitudinal designs are necessary to confirm the temporal sequence of these physical qualities. Second, the sample size, while comparable to numerous field-based performance studies, is relatively modest for structural modeling, which may affect the stability of certain model fit indices. Monte Carlo simulation guidelines generally recommend a minimum of N ≥ 200 for stable SEM estimation [38], and the present sample of N = 86 falls below this threshold. As a result, the SEM findings should be interpreted as exploratory and confirmatory only at a preliminary level, rather than as definitive evidence of the proposed hierarchical structure. Replication with larger, more representative samples is warranted before stronger conclusions can be drawn. Third, study participants are limited to adolescent athletes; therefore, the generalizability of findings to adult or elite-level athletes remains uncertain. Fourth, biological maturation was not directly assessed in the present study. Because explosive power, sprint performance, and COD ability are affected by maturation during adolescence, the observed relationships may partly reflect maturity-related variation rather than purely performance-specific factors. Fifth, COD is evaluated using a pre-planned 505 test that does not account for reactive agility or perceptual decision-making. Thus, the results should be interpreted as COD speed under controlled conditions, not sport-specific reactive agility. Sixth, the fixed testing order, despite standardization, may have introduced sequencing or fatigue effects. Although rest periods were provided, such effects cannot be completely ruled out and may have influenced outcomes. Finally, the sample comprises athletes from different sports disciplines. While this approach increases ecological validity, it may introduce heterogeneity in sport-specific demands and training exposure, including differences in movement patterns, tactical demands, coordination requirements, and accumulated training experience, which may influence COD performance and the observed relationships.

Future research should consider sport-specific analyses to determine whether the hierarchical relationships identified in this study vary across athletic populations. Longitudinal or intervention studies are required to clarify further the causal pathways linking explosive power, sprint performance, and COD ability. Additionally, the present study did not include detailed biomechanical or kinetic measurements (e.g., ground reaction forces, braking forces, joint kinematics, or movement technique variables). As such, the underlying mechanical determinants of sprint and COD performance could not be examined directly, which limits the mechanistic interpretation of the observed relationships, particularly with respect to force-application strategies and movement execution during COD tasks. Future research should integrate biomechanical and technical execution analyses to provide a more comprehensive understanding of COD performance beyond force production alone.

Practically, the findings suggest that training programs aimed at improving COD performance should extend beyond general sprint enhancement and target specific performance components. Particularly, coaches and practitioners may consider emphasizing acceleration development, especially during the initial steps of sprinting, as well as horizontal force production capacity. Furthermore, braking mechanics and deceleration control should be incorporated into training, given their importance in effective COD execution. Finally, improving transition efficiency between deceleration and re-acceleration phases may further enhance COD performance, as these phases are critical for rapid directional changes in sport-specific contexts.

## 5. Conclusions

This study’s findings underscore the central role of sprint performance in linking explosive power and COD ability among adolescent athletes. Indirect effect analyses indicate that sprint performance may act as an intermediary variable between explosive power and COD performance, supporting a hierarchical model in which explosive power relates to sprint performance, which subsequently relates to COD ability. Given the modest sample size (N = 86) and the elevated RMSEA (0.196), however, these SEM-based findings should be regarded as exploratory and preliminary rather than conclusive and interpreted as statistical associations consistent with the proposed model rather than confirmed causal pathways.

Sprint performance emerged as the strongest predictor of COD ability, accounting for a substantial proportion of the variance in COD performance (68.4%). While these associations are statistically robust, the present study’s cross-sectional design and modest sample size preclude definitive causal conclusions. These findings underscore the importance of considering sprint performance as a proximal and functionally relevant determinant of COD ability. From a practical perspective, the findings suggest that training interventions to improve COD performance in adolescent athletes may benefit from prioritizing sprint acceleration and horizontal force production in addition to vertical power development.

Collectively, the study preliminary evidence consistent with a speed-related associative pathway between explosive power and COD performance in youth athletes. The observed statistical associations are consistent with a hierarchical model but should not be interpreted as evidence of direct causal mechanisms. However, the study focused primarily on force-related determinants and did not directly assess technical or biomechanical execution during COD tasks; the findings should be interpreted accordingly.

## Figures and Tables

**Table 1:** Descriptive statistics of anthropometric characteristics and physical performance variables.

Variable	Mean ± SD	Min	Max
Height (cm)	168.65 ± 7.69	149.00	185.50
Body mass (kg)	64.46 ± 13.02	44.00	119.90
Body fat percentage (%)	21.60 ± 8.38	10.00	43.60
Skeletal muscle mass (kg)	28.07 ± 5.46	18.20	49.10
CMJ height (cm)	32.85 ± 6.62	22.00	48.00
SJ height (cm)	28.55 ± 5.58	17.30	41.00
10 m sprint time (s)	1.93 ± 0.18	1.59	2.47
20 m sprint time (s)	3.35 ± 0.30	2.85	4.48
505 COD (left) (s)	2.79 ± 0.28	1.51	3.28
505 COD (right) (s)	2.82 ± 0.21	2.44	3.60
505 COD (s)	2.81 ± 0.23	2.06	3.44

Note: Values are presented as mean ± SD. CMJ = countermovement jump; SJ = squat jump; COD = change-of-direction.

**Table 2:** Pearson’s correlation coefficients among lower-body power, sprint performance, and COD ability.

Variable	1	2	3	4	5	6
1. CMJ height (cm)	1.00					
2. SJ height (cm)	0.80 **	1.00				
3. 10 m sprint time (s)	−0.76 **	−0.70 **	1.00			
4. 20 m sprint time (s)	−0.77 **	−0.69 **	0.97 **	1.00		
5. 505 COD (left) (s)	−0.65 **	−0.63 **	0.63 **	0.67 **	1.00	
6. 505 COD (right) (s)	−0.68 **	−0.66 **	0.74 **	0.82 **	0.77 **	1.00

Note: Values represent Pearson’s correlation coefficients; CMJ = countermovement jump; SJ = squat jump; COD = change-of-direction; negative correlations indicate that greater jump performance is associated with shorter sprint and COD times (i.e., better performance); ** *p* < 0.01.

**Table 3:** Multiple regression analysis predicting COD performance.

Predictor	B	SE	Β	t	*p*
Intercept	1.571	0.402	—	3.906	<0.001
20 m sprint (s)	0.461	0.088	0.58	5.258	<0.001
CMJ (cm)	−0.009	0.004	−0.26	−2.410	0.018

Note: CMJ = countermovement jump. R^2^ = 0.636; adjusted R^2^ = 0.626. Variance inflation factor (VIF) values were 2.44 for both predictors, indicating no evidence of multicollinearity.

**Table 4:** Standardized path coefficients from SEM.

Path	β	*p*
Power → sprint	−0.82 ***	<0.001
Sprint → COD	0.62 ***	<0.001
Power → COD	−0.28 *	0.043

Note: β = standardized path coefficient; COD = change-of-direction ability. * *p* < 0.05, *** *p* < 0.001.

## Data Availability

The data supporting the findings of this study are available within the article.
